# Recommendations for the management of autoinflammatory diseases

**DOI:** 10.1186/1546-0096-13-S1-P133

**Published:** 2015-09-28

**Authors:** N ter Haar, M Oswald, J Jeyaratnam, J Anton, K Barron, P Brogan, L Cantarini, C Galeotti, G Grateau, V Hentgen, M Hofer, T Kallinich, I Kone-Paut, H Lachmann, H Ozdogan, S Ozen, R Russo, A Simon, Y Uziel, C Wouters, B Feldman, B Vastert, N Wulffraat, S Benseler, J Frenkel, M Gattorno, J Kuemmerle-Deschner

**Affiliations:** 1 University Medical Center Utrecht, Utrecht, Netherlands; 2 University Hospital Tuebingen, Tuebingen, Germany; 3 Hospital Sant Joan de Déu, University of Barcelona, Barcelona, Spain; 4 National Institute of Health, Bethesda, USA; 5 UCL Institute of Child Health, London, UK; 6 Policlinico Le Scotte, University of Siena, Siena, Italy; 7 Reference Centre for Autoinflammatory Disorders CEREMAI, Bicêtre Hospital, Paris, France; 8 Hôpital Tenon, University Pierre-et-Marie-Curie, Paris, France; 9 French Reference Centre for Auto-Inflammatory Diseases in Children, Centre Hospitalier de Versailles, Le Chesnay Cedex, France; 10 University of Lausanne, Lausanne, Switzerland; 11 Charité University Medicine, Berlin, Germany; 12 UCL Medical School, London, UK; 13 Cerrahpasa Ic Hastaliklari Klinigi, Universtity Istanbul, Istanbul, Turkey; 14 Hacettepe University Faculty of Medicine, Ankara, Turkey; 15 Hospital de Pediatría Garrahan, Buenos Aires, Argentina; 16 Radboud University Medical Center, Nijmegen, Netherlands; 17 Meir Medical Center, Tel-Aviv University, Tel-Aviv, Israel; 18 University Hospital Leuven, Leuven, Belgium; 19 The Hospital for Sick Children, Toronto, Canada; 20 Alberta Children's Hospital, University of Calgary, Calgary, Canada; 21 G. Gaslini Institute, Genoa, Italy

## Background

Autoinflammatory diseases are rare disorders that lead to significant morbidity and mortality. Due to the low patient numbers, evidence-based guidelines are lacking and management is mostly based on physician's experience. In 2012, a European initiative called SHARE was launched to optimize and disseminate diagnostic and management regimens in Europe for children and young adults with rheumatic diseases. One of the aims of SHARE was to provide evidence-based recommendations for the management of the autoinflammatory diseases Cryopyrin-Associated Periodic Syndromes (CAPS), Tumor necrosis factor Receptor Associated Periodic Syndrome (TRAPS) and Mevalonate Kinase Deficiency (MKD).

## Methods

Evidence-based recommendations were developed using the European League Against Rheumatism (EULAR) standard operating procedure. An expert committee of paediatric and adult rheumatologists was convened. Recommendations derived from the systematic literature review were evaluated by an online survey and subsequently discussed at a consensus meeting using Nominal Group Technique. Recommendations were accepted if more than 80% agreement was reached.

## Results

In total, four overarching principles, six recommendations on diagnosis, twenty recommendations on therapy and twelve recommendations on monitoring were accepted with ≥80% agreement among the experts. Topics include (but are not limited to) the use of validated scores for diagnosis and disease activity, therapy with biologicals, NSAIDs and corticosteroids, and items to assess in monitoring of a patient.

## Conclusion

The SHARE initiative provides recommendations for the management of the autoinflammatory diseases CAPS, TRAPS and MKD.

